# Nutritional Quality of Four Novel Porridge Products Blended with Edible Cricket (*Scapsipedus icipe*) Meal for Food

**DOI:** 10.3390/foods11071047

**Published:** 2022-04-05

**Authors:** Nelly C. Maiyo, Fathiya M. Khamis, Michael W. Okoth, George O. Abong, Sevgan Subramanian, James P. Egonyu, Cheseto Xavier, Sunday Ekesi, Evanson R. Omuse, Dorothy Nakimbugwe, Geoffrey Ssepuuya, Changeh J. Ghemoh, Chrysantus M. Tanga

**Affiliations:** 1 International Centre of Insect Physiology and Ecology (icipe), P.O. Box 30772, Nairobi 00100, Kenya; nmaiyo@icipe.org (N.C.M.); fkhamis@icipe.org (F.M.K.); ssubramania@icipe.org (S.S.); pegonyu@icipe.org (J.P.E.); cxavier@icipe.org (C.X.); sekesi@icipe.org (S.E.); evansonomuse12@gmail.com (E.R.O.); 2 Department of Food Science, Nutrition and Technology, University of Nairobi, P.O. Box 30197, Nairobi 00100, Kenya; mwokoth@uonbi.ac.ke (M.W.O.); ooko.george@uonbi.ac.ke (G.O.A.); 3 Department of Food Technology and Nutrition, School of Food Technology, Nutrition and Bioengineering, Makerere University, Kampala P.O. Box 7062, Uganda; dnakimbugwe@gmail.com (D.N.); gksepuya@gmail.com (G.S.); 4 Centre for African Bio-Entrepreneurship (CABE), P.O. Box 25535, Nairobi 00603, Kenya; janiceghemoh@yahoo.com

**Keywords:** underutilized food resources, grain amaranth, edible cricket meal, complementary porridge, nutrient quality, malnutrition and food security

## Abstract

Currently, no data exist on the utilization of the newly described cricket species (*Scapsipedus icipe*) meal as additive in food products, though they have high protein (57%) with 88% total digestibility as well as a variety of essential amino acids. This article presents the first report on the effects of processing techniques and the inclusion of cricket meal (CM) on the nutrient and antinutrient properties of four porridge products compared to a popularly consumed commercial porridge flour (CPF). Porridge enriched with CM had significantly higher protein (2-folds), crude fat (3.4–4-folds), and energy (1.1–1.2-folds) levels than the CPF. Fermented cereal porridge fortified with CM had all three types of omega-3 fatty acids compared to the others. The vitamin content across the different porridge products varied considerably. Germinated cereal porridge with CM had significantly higher iron content (19.5 mg/100 g). Zinc levels ranged from 3.1–3.7 mg/100 g across the various treatments. Total flavonoid content varied significantly in the different porridge products. The phytic acid degradation in germinated and fermented porridge products with CM was 67% and 33%, respectively. Thus, the fortification of porridge products with cricket and indigenous vegetable grain powder could be considered an appropriate preventive approach against malnutrition and to reduce incidences in many low-and middle-income countries.

## 1. Introduction

The malnutrition of children in sub–Saharan African (SSA) countries is a serious health concern [1,2]. Protein-energy malnutrition (PEM) and deficiencies of micronutrients including iron, zinc, and vitamin A are the most common forms of malnutrition reported in these countries [3,4]. Kenya is amongst the 20 countries accounting for 80% of the world’s malnourished children [5] where stunting, wasting, and underweight in children below five years have been estimated at 26%, 4%, and 11%, respectively [6]. Malnutrition in infancy and early childhood affects physical growth and cognitive behaviour, leading to delays in mental and motor development, as well as increased morbidity and mortality [7]. Low socio-economic status is considered the key underlying cause where children lack food or survive on diets of low nutritional quality [8]. 

The African continent is blessed with a rich diversity of food crops, most of which have received little or no attention in terms of research and development of policy frameworks that could promote their effective commercial and industrial utilization. Grain amaranth (*Amaranthus* spp.) and finger millets are some of such neglected and underutilized species that could be used to produce porridge products to serve as important traditional beverages and complementary food for adults and children, respectively, of all ages in Africa [9,10]. Despite these cereals being rich in carbohydrates, their energy and nutrient densities are extremely low [11], partly due to the presence of anti-nutritional factors that restricts the bioavailability of essential nutrients [12]. Finger millet grain contains high amounts of proteins and minerals compared to other staple cereals [13] and is the most preferred grain in composite flour for making porridge [1]. Amaranth (*Amaranthus* spp.) is an indigenous African leafy vegetable grown in at least fifty tropical countries and consumed by several million people [2,14] for many nutritional reasons. Farmers in sub-Saharan Africa cultivate amaranth either for its leaves or for its grain [14]. The leaves are rich in vitamin C and pro-vitamin A as well as in iron, zinc, and calcium [15]. The grains are also rich in quality protein, lysine, and calcium and are consumed directly or used to fortify maize flour [16,17]. Amaranth grain contains bioactive compounds with health promoting effects [18]. Studies have shown that regular consumption of amaranths has the potential [18] to reduce cholesterol levels [19], benefit people suffering from hypertension and cardiovascular disease [20,21], improve liver functions [22,23], and prevent cancers [24]. The ability health benefits of amaranth are due to the bioactive compounds such as protocatechuic, hydroxybenzoic, caffeic and ferulic acids, rutine, nicotiflorin, and isoquercetin present in it [25]. While amaranth is being widely cultivated in Africa as a vegetable, its grains have been documented to contain excellent nutritional properties and have been milled and blended with flours of other cereal staples to improve their overall acceptability [26]. However, these grains contain a high content of anti-nutrients, mostly in the form of phytic acid [27,28]. These anti-nutrients can be reduced to improve the nutritional quality through traditional food processing methods, including soaking, germination, fermentation, roasting, and milling [13].

The use of edible insect meal to fortify porridge products has received limited research attention, even though it has been postulated to contain high-quality nutrients, which are easily digestible and more bio-available than those available from plant and animal food sources [29,30]. This will contribute to the Sustainable Development Goal (SDG) 2 of zero hunger championed by the United Nations, which aims at improving food and nutritional security [29]. Food-to-food fortification of complementary foods with nutrient-rich ingredients like edible insects that are rich in protein, amino acids, fatty acids, vitamins, and minerals (e.g., zinc, iron, and calcium) [30,31,32,33] can help in the attainment of this goal. Thus, cricket consumption could be an immediate solution to many of the nutrient deficiency issues and could be both an inexpensive and effective option. The use of naturally available food resources such as insects like crickets, would easily be favoured by policy makers in view of their sustainability [34,35,36]. Murugu et al. [37] have demonstrated that the newly described cricket *Scapsipedus icipe* Hugel and Tanga is significantly rich in protein (57%), fat (36%), amino acids, minerals, and vitamins. However, no information exists on their inclusion into cereal-based human food products, although there are promising indications that they can significantly improve the nutritional quality of food products. 

Furthermore, edible insects are commonly processed using different techniques (frying, drying, roasting, smoking, boiling, toasting, and steaming) to ensure microbial safety, increase shelf-life, and improve on the sensory appeal [4,7,37,38]. The utilization of appropriate processing methods is critical to mitigate spoilage [5]. Several studies have shown that processing methods can cause significant losses and degradation of essential nutrients besides enhancing their levels, digestibility, and bioactivity of the others [3,6,7], either through solubilization, leakages, and intra- and inter-biochemical reactions. Ssepuuya et al. [8] reported the effects of two thermal processes on the nutritional composition, colour, and aroma compounds of *R. differens*. Furthermore, investigation by Nyangena et al. [4] apprised the effects of toasting, boiling, and drying techniques on proximate composition and microbial quality of *R. differens* and other edible insects only. None of these studies looked at the potential effects of processing techniques and mixing of amaranth integrated with cricket meal on compounded human food products. This study sought to bridge this gap by comparing the effects of four common food processing methods of mixing of amaranth, cricket meal, and finger millets into porridge products on the nutrient (proximate composition, fatty acids, and minerals) and antinutrient composition as well as bioactive compounds (total flavonoid content and vitamins). This detailed report on the effect of the different processing methods applicable to cereal-based porridge food products with cricket meal is inevitable, especially with recent findings revealing the significance of edible insects and amaranth in the fight against malnutrition and food insecurity [9]. The campaign for consumption of insects, grains, and vegetables for the maintenance of good health has become ever more popular globally [29,30,39].

## 2. Materials and Methods

### 2.1. Raw Materials

Grain amaranth (*Amaranthus cruentus*) and finger millet (*Eleusine coracan* L.) were purchased from a local market in Nairobi, Kenya. One of the most traded brands of commercial porridge flour (CPF) (FAMiLA*^®^* pure WIMBI porridge, Unga Limited, Nairobi, Kenya) was purchased from a supermarket in the Kenyan capital, Nairobi. The unique formulation of FAMiLA*^®^* consists of pure finger-millet flour fortified with minerals (calcium), carbohydrates, and proteins (particularly lysine). This product was chosen because it is popular and consumed by adults and children of all ages. On the other hand, edible crickets (*Scapsipedus icipe*) were obtained from the Animal Rearing and Containment Unit (ARCU) at the International Centre of Insect Physiology and Ecology (*icipe*), Nairobi, Kenya.

### 2.2. Preparation of Raw Materials

#### 2.2.1. Preparation of Crickets

Frozen cricket samples were allowed to thaw overnight at a 5 °C refrigerated temperature. The samples were then washed thrice in fresh, clean tap water at 18 °C to remove dirt, drained, and appropriately heat-treated in hot water at 100 °C for 5 min for efficient sterilization. Thereafter, the crickets were oven-dried (WTB binder, Tuttlingen, Germany) at 60 °C for 24 h, milled using an electric grinder [Medical Research Council (MRC) laboratory grinder, London, UK], and sieved through a 0.595-mm aperture sieving mesh. The resulting powder was packaged in a sterile zip-lock polyethylene bag and stored at 4 °C for subsequent composite flour formulation.

#### 2.2.2. Processing of Finger Millet and Amaranth Grains

Finger millet and amaranth grains were each divided into a batch of 500 g in four replications. The first batch was germinated according to the method described by Onyango et al. [40]. Briefly, the grains were cleaned and steeped in tap water for 24 h at 25 °C and germinated for 72 h at 25 °C in the dark, while being moistened and turned at 12–hour intervals. Germination was halted by oven-drying at 50 °C for 12 h. The dried germinated grains were then removed and milled using an electric grinder (MRC laboratory grinder, London, UK). The second batch was roasted according to the modified method [41]. The grains were spread in a uniform, thin layer on an oven tray and roasted in a pre-heated oven at 120 °C for 20 min. The grains were allowed to cool to room temperature before milling. The third batch was milled and fermented according to the method described by Onyango et al. [10] with slight modifications. The composite flour was mixed with tap water that had been boiled, then cooled to 45 °C, at a ratio of 2:3 (flour to water). The slurries were fermented spontaneously in round-bottomed flasks placed in a water bath (Blue M Electric Company, IL 60406, USA) at 45 °C. After 24 h the fermented slurries were inoculated into freshly prepared slurries before fermenting at 45 °C for 24 h. The samples were then spread on trays and dried in an oven at 50 °C for 24 h and ground in an electric grinder (MRC laboratory grinder, London, UK). The fourth batch was unprocessed grains treated according to farmers’ field practices of open sun drying and storage, using appropriate technology to ensure availability throughout the year [42]. Thereafter, the grains were milled and prepared for further processing into porridge products. The fifth batch was considered as the “control treatment” and consisted of the CPF, which had undergone approved commercial standards of processing. All the samples were ensured to have a moisture content of <12.0%.

### 2.3. Porridge Flour Formulations

Composite flour formulations were developed and optimized using the Linear Programming of the Excel solver 2010 version, based on the minimum recommended dietary allowance (RDA) for protein (13 g), energy (500 kcal), calcium (500 mg), iron (7 mg), and zinc (4.1 mg) for children aged 1–3 years [11,12]. Porridge formulations were prepared by mixing flours of finger millet, amaranth, and cricket, at a ratio of 60:30:10. The porridge mix samples were coded as follows: commercial porridge flour (CPF), fermented finger millet − amaranth + cricket (FFM–AC), germinated finger millet − amaranth + cricket (GFM–AC), roasted finger millet − amaranth + cricket (RFM–AC), and unprocessed sundried finger millet − amaranth + cricket (UFM–AC). These new products supplemented with cricket meal and amaranth were compared with a commercial porridge product widely used by millions of households in East Africa.

### 2.4. Analysis of Proximate Composition

Proximate analysis for the flour was determined following the Association of Official Analytical Chemists (AOAC) methods [13] in three replications. Moisture content was determined in an air oven adjusted to 105 °C (method 925.10). The Kjeldahl method (method 978.04) was used to assess the crude protein (N × 6.25). Fat content was extracted using petroleum ether in a Soxhlet extractor (method 930.09). Ash content was determined by gravimetries (method 930.05). Fibre was determined by acid digestion and loss of ignition (method 930.10). Carbohydrate contents were determined as the difference (CHO% = [100−protein %−fat %−crude fibre %−ash %]). The energy value was computed by multiplying the Atwater factors of 4, 9, and 4 with protein %, fat % and carbohydrate % contents, respectively.

### 2.5. Determination of Mineral Composition

Mineral composition was established in three replications by the ICP OES quantitation method as follows. Exactly 0.5 g of each sample was digested with concentrated HNO_3_ (8 mL) and 30% H_2_O_2_ (2 mL) and left overnight in a fume chamber. Samples were then digested in a temperature-controlled block digester (Model TE007—A, TECNAL, São Paulo, SP, Brazil) following these conditions: 75 °C for 30 min, 120 °C for 20 min, 180 °C for 20 min, and 200 °C for 10 min. Resulting solutions were cooled and transferred to 25 mL falcon tubes and diluted with 2% nitric acid. Mineral compositions were assessed using an inductively coupled plasma optical emission spectrometer (ICP OES) (Model Optima 2100 DV Perkin Elmer, MA, USA) and analysed using WinLab 32 software (Perkin Elmer, Poway, CA 92064, USA). The following operational conditions were used: radiofrequency power (1.45 kW), auxiliary gas flow rate (1.5 L min^−1^), plasma gas flow rate (15.0 L min^−1^), nebuliser gas flow rate (0.7 L min^−1^), sample flow rate (1.5 L min^−1^), source equilibrium time (10 s), and delay time (10 s). Signal intensity measurements of the analytes in all samples solutions were carried out at wavelengths (nm) as follows: Mg: 285.213, Fe: 259.939, Mn: 257.61, Ca: 317.933, P: 213.617, Mo: 202.031, K: 766.49, Al: 396.153, Cu: 224.7, Co: 228.616, and Zn: 213.857. ICP OES quantification was carried out using a multi-element standard solution (TraceCERT) CatNo.43843 (Sigma-Aldrich, Saint Louis, MO, USA). The calibration standards were prepared by titrating the standard solution in 2% (*v*/*v*) nitric acid to obtain the working ranges required (400–4000 µg L ^−1^). The correlation coefficient obtained was ≥0.999. Calibration was performed using WinLab 32 software (Perkin Elmer, Poway, CA 92064, USA).

### 2.6. Assessment of Fatty Acids

#### 2.6.1. Folch Extraction Method

Oil extraction from the formulated porridge flours was achieved based on the modified method [43]. In three replications, 5 g each of sample were diluted with 10 mL of 2:1 dichloromethane (DCM) and methanol (MeOH). The mixtures were vortexed for 1 min, sonicated for 10 min, and centrifuged at 4200 rpm for 10 min. The supernatants were filtered using grade 1 and 90 mm (diameter) Whatman filter paper into a 250 mL round bottomed flask and evaporated in vacuo to yield approximately 200 mg of oil.

#### 2.6.2. Fatty Acid Determination

Compositions of fatty acid (FA) in the oil extract from formulated porridge flours were examined as fatty acid methyl esters (FAMEs), according to the modified method [27]. Approximately 1 mL of sodium methoxide solution (100 mg/mL) was added to 100 mg of recovered oil extract, vortexed for 1 min, followed by sonication for 10 min. The resulting mixture was placed in a water bath (70 °C) for 1 h and the reaction halted by adding of 100 µL deionized water, followed by vortexing for another 1 min. To extract the FAMEs, 1 mL of gas chromatography (GC)-grade hexane (Sigma-Aldrich, St. Louis, MO, USA) was added to the mixture, followed by 20 min centrifugation at 14,000 rpm. The resulting hexane layer (upper) was dried over anhydrous Na_2_SO_4_, and analysis was performed using an Agilent GC–MS on a 7890A GC, connected to a 5975 C mass selective detector (Agilent Technologies Inc., Santa Clara, CA, USA). GC was fitted with a (5%–phenyl)–methylpolysiloxane (HP5 MS) low bleed capillary column (30 m × 0.25 mm i.d., 0.25 µm (J&W, Folsom, CA, USA). The sample injection volume was 1 µL. Helium acted as the carrier gas at flow rates of 1.25 mL/min. The oven temperature was programmed between 35 to 285 °C, with the initial and final temperature kept for 5 and 20.4 min, respectively, with a rising rate of 10 °C minute^−1^. An ion trap mass selective detector was maintained at the ion source temperature of 230 °C and quad temperature of 180 °C. The mass detector was run in electron impact (EI) mode (70 eV). Fragment ions were determined in the full scan mode at over 40–550 m/z mass range. The filament was delayed for 3.3 min. Authentic standard methyl octadecenoate (0.2–125 ng/μL) serial dilutions prepared from octadecanoic acid (≥95% purity) (Sigma-Aldrich, St. Louis, MO, USA) were analysed using GC-MS in full scan mode. GC-MS generated a linear calibration graph of peak area versus concentration with the equation; [*y* = 5*E* + 0.7*x* + 2*E* + 0.7] with R^2^ of 0.9997. This regression equation was used for the external quantification of the different FA and sterols from the samples.

A Hewlett-Packard (HP Z220 SFF intel xeon) workstation with ChemStation B.02.02. data acquisition software (Palo Alto, CA, USA) was used to control the operation of GC-MS. ChemStation integration parameters were calibrated as follows: initial threshold = 3, initial peak width = 0.010, initial area reject = 1, and shoulder detection = on. These calibrations yielded the mass spectrum for the peaks. The identification of compounds were based on the comparison of the generated mass spectra and retention times with those of authentic standards and reference spectra in library-MS databases: National Institute of Standards and Technology (NIST) 05, 08, and 11. The determination of the FAMES in all the flour samples was carried out in triplicates.

### 2.7. Determination of Water-Soluble Vitamins

The determination of water-soluble vitamins was carried out according to Thermo Fisher Scientific [28] (Waltham, MA, USA). For sample preparation, 100 mg of flour sample was mixed with 25 mL of distilled water in a 50 mL falcon tube. The mixture was ultra-sonicated for 15 min and solution filtered through 0.2-µm filters into UPLC vials. The chromatographic analysis was performed on a Liquid chromatographic system with a diode array detector (LC-30AC with Nexera column oven CTO-30A, Shimadzu, Tokyo, Japan). A Phenomenex C18 Column Synergi, 100 × 3.00 mm, 2.6 µm polar (Phenomenex, Torrance, CA, USA) at 30 °C, was used. The mobile phase consisted of two phases: mobile phase A: 25 mM phosphate buffer; mobile phase B: 7:3 *v*/*v* Acetonitrile-Mobile phase A. The total run time was 12 min with a flow rate of 0.4 mL min^−1^. Stock solutions of 1.0 mg/mL were prepared by dissolving the individual water-soluble vitamin standards in distilled water except for Vitamin B_2_ (in 5 mM potassium hydroxide) and Vitamin B_9_ (in 20 mM potassium hydrogen carbonate). Four calibration standards at a concentration of 2, 5, 10 and 15 µg/mL were prepared from the mixed stock solutions. The retention times (mins) for the vitamins were as follows: Vitamin C—1.596, Vitamin B_1_—1.922, Nicotinic acid—2.228, Vitamin B_6_—3.496, Nicotinamide—5.050, Vitamin B_5_—6.772, Vitamin B_9_—8.236, Vitamin B_12_—8.936, and Vitamin B_2_—9.224. R^2^ was ≥0.996. All determinations were carried out in triplicates.

### 2.8. Determination of Fat-Soluble Vitamins

Fat-soluble vitamins was determined according to a method described by Bhatnagar et al. [29]. Briefly, 6 mL of ethanol with 0.1% (BHT)) was added to 500 mg of the flour sample and homogenized. To the resulting mixture, 120 µL of potassium hydroxide 80% (*w*/*v*) was added and vortexed for 1 min followed by incubation at 80 °C for 5 min. Cooling was carried out by placing the test tubes in ice, and 4 mL of deionized water was added to the mixture to enhance phase separation, followed by vortexing for 1 min. For extraction, 5 mL HPLC-grade hexane (Sigma-Aldrich, St. Louis, MO, USA) was added to the mixture, followed by a 5 min centrifugation at 3000 rpm. The resulting hexane layer (upper) was transferred into a separate test tube, and pellet was re-extracted twice more using hexane and the upper phases collected and pooled. Drying was carried out under nitrogen gas flow, and the residue reconstituted in 1 mL of methanol: tetrahydrofuran (85:15 *v*/*v*), vortexed and sonicated for 30 s and filtered into HPLC sample vials. Analysis was performed using reverse-phase HPLC (Shimadzu, Tokyo, Japan) linked to SPD-M2A detector. The UPLC was fitted with a YMC C30, carotenoid column (3 µm, 150X3.0 mm, YMC Wilmington, NC, USA). The mobile phase consisted of two phases: A: methanol/tert-butyl methyl ether/water (85:12:3, *v*/*v*/*v*, with 1.5% ammonium acetate in the water) and B: methanol/tert-butyl methyl ether/water (8:90:2, *v*/*v*/*v*, with 1% ammonium acetate in the water). The injection volume was 10 μL with a total flow rate of 0.4 mL/min. The retention times for retinol and α- and γ-tocopherol were 2.74, 5.40, and 6.29 min, respectively. Compounds presenting the eluting sample were monitored at 290 nm. Peaks were identified by their retention time, and absorption spectra were compared to those of known standards (Sigma Chemicals). Sample concentrations were calculated by comparing peak area of samples to peak area of the standards.

### 2.9. Assessment of Phytic Acid, Tannins, and Flavonoids

Phytic acid contents were determined using a K–PHYT Phytic Acid (Phytate)/Total Phosphorus kit (Megazyme Int. Ireland Ltd., Wicklow, Ireland) following the manufacturer’s instructions [30]. A flour sample (1 g) was added to 20 mL of 0.8 M HCl and mixed by shaking at room temperature overnight, and 1.5 mL of the resulting extract was centrifuged at 13,000 rpm for 10 min. The supernatant (0.5 mL) was neutralized with 0.5 mL of 0.8 M NaOH and used for the enzymatic dephosphorylation reaction and the subsequent total phosphorous and inorganic phosphate quantification. In parallel, 0.5 mL of the neutralized sample were used to quantify the inorganic phosphate of the sample. After the enzymatic treatment, the total phosphorous and total inorganic phosphate of the samples were used for colorimetric development to estimate the phosphorous content. This kit measures the inorganic phosphate released from the extracted flour sample after treatment with phytase and alkaline phosphatase. The free inorganic phosphate content was estimated from samples not treated with phytase. Experiments were performed in triplicate.

Total tannin content was determined by Folin–Denis method, using a microplate reader as outlined by Saxena et al. [31]. The results were expressed in mg tannic acid equivalents (TAE)/100 g of sample. The total flavonoid content was analysed using the Aluminium Chloride colorimetric [32]. The results were expressed in mg catechin equivalents (CEQ)/100 g of dry sample. All determinations were carried out in triplicates.

**Figure 1:**
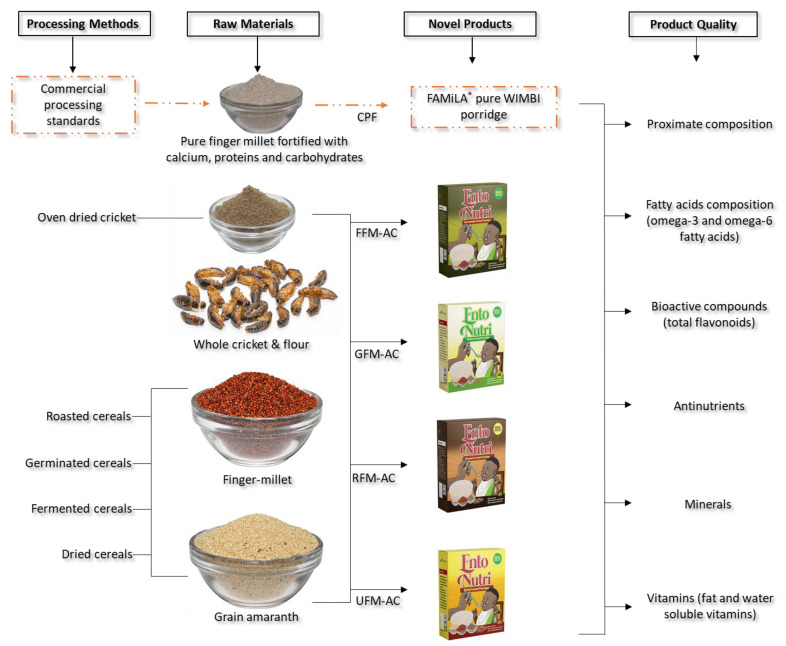
Flow diagram showing the raw materials (grain amaranth, finger-millet, and cricket) and the formulated porridge products [CPF = commercial porridge flour; FFM–AC = fermented finger millet − amaranth + cricket; GFM–AC = germinated finger millet − amaranth + cricket; RFM–AC = roasted finger millet − amaranth + cricket; and UFM–AC = unprocessed finger millet − amaranth + cricket.

A flowchart of the various processes involved throughout the development of the novel porridge products and nutrient profiling are summarized in Figure 1 below.

### 2.10. Determination of Molar Ratios

Mineral bioavailability (zinc, iron, and calcium) was expressed as a phytate/mineral molar ratio [33]. Moles of phytic acid were calculated by dividing the recorded value of phytic acid with its atomic weight (660), while the moles of minerals (zinc, iron, and calcium) were determined by dividing the recorded values by the individual molecular weight of the respective compounds (i.e., Zn = 65, Fe = 56, and Ca = 40).

### 2.11. Statistical Analysis

Datasets of proximate compositions, mineral contents, fatty acids (FAs), and anti-nutritional data were subjected to analysis of variance (ANOVA). The means were separated using the HSD Tukey tests. Statistical analysis was performed using R Studio software version 1.3.1093-1(Boston, MA, USA) [34].

## 3. Results

### 3.1. Proximate Composition

The results of the proximate composition and energy values of the formulated porridge products, which consisted of grain amaranth, finger-millet, and cricket, are presented in Table 1. Samples varied significantly (*p* < 0.05) in their proximate values, except on ash content. All products enriched with cricket had significantly (*p* < 0.05) higher protein (2-fold), fat (3.4–4-fold), and energy content (1.1–1.2-fold) when compared to the commercial porridge flour. The germinated sample presented the highest protein content (16.12 g/100 g) followed by the unprocessed sample (15.9 g/100 g), whereas the fat content of the fermented product (7.2 g/100 g) was significantly (*p* < 0.05) lower than that of other formulated products (8.1 g–8.31 g/100 g), but higher when compared to the commercial porridge flour (2.4 g/100 g). The highest crude fibre content was recorded in the germinated sample (5.3 mg/100 g), whereas the lowest was recorded in the fermented sample (3.3 mg/100 g).

### 3.2. Fatty Acids

**Figure 2:**
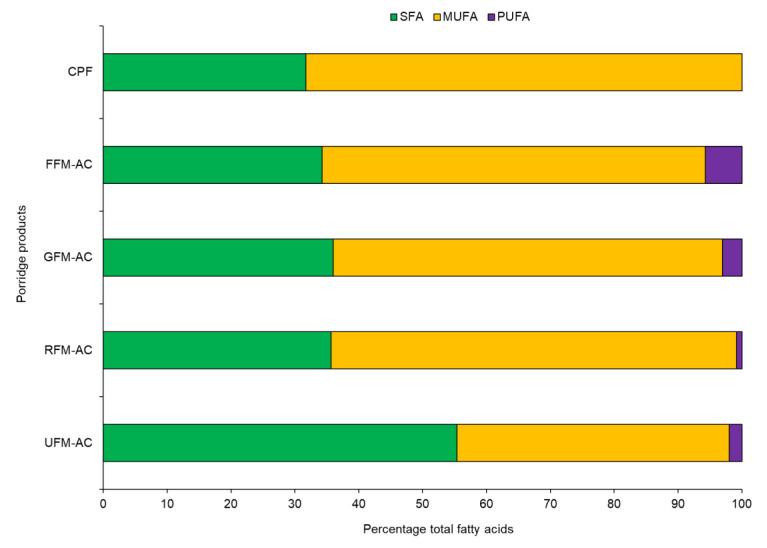
Total fatty acid composition in different porridge flour samples. SFA = Saturated fatty acid; MUFA = monounsaturated fatty acid; PUFA = polyunsaturated fatty acid (PUFA); FFM–AC = fermented finger millet − amaranth + cricket; RFM–AC = roasted finger millet − amaranth + cricket; GFM–AC = germinated finger millet − amaranth + cricket; UFM–AC = unprocessed finger millet − amaranth + cricket; CPF = commercial porridge flour.

The results of FAMEs in different porridge flour samples are presented in Table 2. Of the 44 FAs detected, the proportion of saturated fatty acids (SFAs), monounsaturated fatty acids (MUFAs), and polyunsaturated fatty acids (PUFAs) present in porridge flour samples were 23%, 54%, and 23%, respectively. More FAMEs (3 to 4-fold) were detected in the cricket enriched porridge flours than in the commercial porridge flour. Additionally, the proportion of each group of FAMEs (SFA, MUFA, and PUFA) across the different porridge products are illustrated in Figure 2. Of the 24 SFAs detected, Methyl Hexadecanoate (palmitic acid) contributed the highest proportion, followed by Methyl Octadecanoate (stearic acid), across the flour samples. In addition, Methyl 9E-Octadecenoate (oleic acid) was the predominant MUFA, whereas Methyl 9Z,12Z-Octadecadienoate (linoleic acid, LA) accounted for the highest proportion of the PUFAS.

The proportion of omega-3 fatty acids, namely α-linolenic acid (ALA), Eicosapentaenoic acid (EPA), Alpha Eleostearic acid (α-ESA), and Docosapentaenoic acid (DHA) differed considerably across the flour samples. α-linolenic acid (ALA) was detected in all the cricket enriched samples, while EPA was present in the fermented and germinated sample. DHA was only present in the fermented flour. On the effect of processing, PUFAs increased significantly (*p* < 0.05) by 30% during fermentation and decreased by 3% during roasting. Additionally, the roasting process caused a significant (*p* < 0.05) increase in both MUFAs and SFAs by 27% and 10%, respectively. Germination caused a slight increase in 9% and 12% in both MUFAs and PUFAs, respectively. The ratio of omega-6 to omega-3 varied significantly among the different flour samples and ranged from 3.2 to 4.4.

### 3.3. Vitamin Content

The results show significant variations in the vitamin content (Table 3). Cricket enriched formulations had a significantly (*p* < 0.05) higher content of vitamin B_12_, vitamin B_5_, B_6_, nicotinamide, and thiamine. Thiamine content ranged from 4.3–39.5 mg/100 g while vitamin B_12_ was in the range of 3.2–37.7 mg/100 g. Processing methods had a significant (*p* < 0.05) effect on the vitamin levels. Germinated and fermented samples had enhanced levels of vitamin C, nicotinamide, vitamin B_5_, folate, and vitamin B_6_. However, there was a significant (*p* < 0.05) decrease to undetectable levels in the contents of nicotinic acid and thiamine during fermentation and germination, respectively. Vitamin B_12_ showed significant reductions in all the processed porridge flours, while riboflavin content was not affected by processing. The roasting process had a negligible effect on vitamins, except for significant (*p* < 0.05) reductions in nicotinic acid and vitamin B_12,_ and a slight reduction in vitamin C. The retinol and α- and γ-tocopherol content differed significantly (*p* < 0.05) in different porridge flour samples. Retinol content increased slightly during fermentation, while germination process decreased the levels of retinol and increased the α-tocopherol content. However, the roasting process did not affect the levels of α- and γ-tocopherol.

### 3.4. Mineral Content and Molar Ratios

The mineral content varied significantly across the porridge flour products as shown in Table 4. The iron content of the formulated flours ranged between 8.59–19.48 mg/100 g with the germinated sample having the highest content (19.48 mg/100 g). Zinc content was in the range of 3.08–3.70 mg/100 g, while the range obtained for calcium was from 234.87 mg–278.61 mg/100 g. The calcium content of the commercial porridge flour was significantly (*p* < 0.05) higher at 312.69 mg/100 g when compared to the formulated samples, while the Zn content was significantly lower (1.86 mg/100 g). The fermented sample had significantly (*p* < 0.05) lower levels of magnesium and phosphorous, but the levels of calcium, copper, iron, and zinc did not vary when compared with the unprocessed formulation. Calcium and iron content in the germinated sample were higher than in other formulations. The roasting process did not significantly (*p* > 0.05) affect the mineral content.

Processing methods had a significant (*p* < 0.05) influence on the phytate/mineral molar ratios (Table 4). The critical values for the Phy:Fe, Phy:Zn, and Phy:Ca ratios were 1.0, 15.0 and 0.24, respectively [35]. Germinated sample had the lowest ratios for Phy:Fe (1.31) and Phy:Zn (7.43), whereas commercial flour had the lowest ratio for Phy:Ca (0.03). Phy:Ca ratios recorded in all the flours were below the limit threshold, except for the roasted sample (0.24) which had the highest values of all molar ratios.

### 3.5. Phytic Acid, Tannins, and Flavonoids

**Figure 3:**
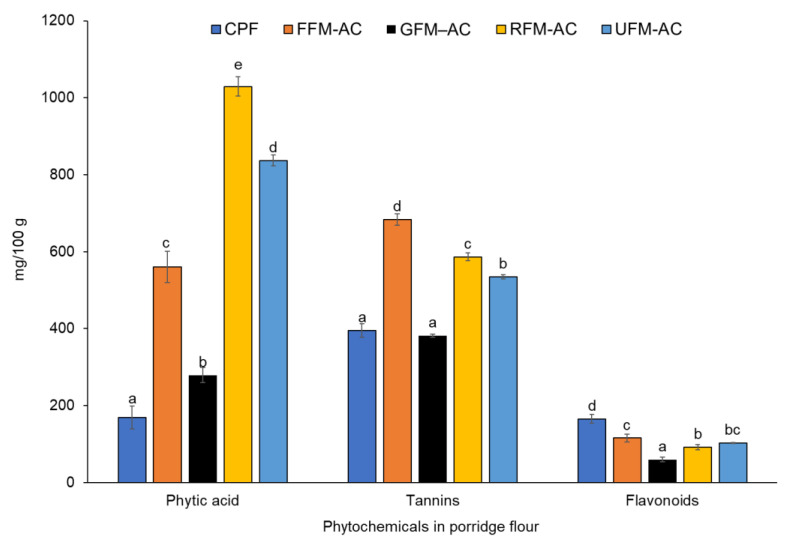
Bar graphs represent mean and error bar standard deviation of phytochemical content in the flour samples. Different letters above the error bar indicate significant differences in phytochemical content. CPF = commercial porridge flour; FFM–AC = fermented finger millet − amaranth + cricket; GFM–AC = germinated finger millet − amaranth + cricket; RFM–AC = roasted finger millet − amaranth + cricket; and UFM–AC = unprocessed finger millet − amaranth + cricket. mg tannic acid equivalents (TAE)/100 g of sample, mg catechin equivalents (CEQ)/100 g of dry sample.

The phytic acid, tannin, and flavonoid contents of the flour samples are summarized in Figure 3. The phytic acid content of the roasted sample was significantly (*p* < 0.05) higher (1029 mg/100 g), followed by the unprocessed sample (837 mg/100 g), while the germinated sample had the lowest (279 mg/100 g) among the processed formulations. The highest (*p* < 0.05) tannin content (683 mg/100 g) was recorded in the fermented sample and the lowest content (381 mg/100 g) was detected in the germinated sample. The flavonoid content of the porridge flours was in the range of 60 mg–166 mg/100 g, with the highest value (166 mg/100 g) recorded for the commercial porridge flour.

## 4. Discussion

In low- and middle-income countries, child malnutrition contributes to about 45% of under-five child mortality, and this portends great danger to Africa’s growth and development. One third of child deaths in Africa are attributable largely to protein energy malnutrition and micronutrient deficiencies [44,45], which could be solved by exploring underutilized nutritious crop and animal species of African origin. Traditional cereal-based porridge preparation has been considered as one of the major causes of protein energy malnutrition in developing countries [35]. The traditional complementary foods are usually low in protein and energy density, while containing high anti-nutrient content [36]. This study shows that formulating porridge products with underutilized edible cricket and amaranth meal, using household techniques to increase the protein, energy, vitamin, fatty acids, and mineral content in the porridge flour, could be the best substitute for traditional weaning flours. The recommended daily intake of protein in children aged 1–3 years is 13 g/day [12], and our study showed that the protein content in cricket and amaranth enriched flour ranged between 15.34–16.12 g/100 g (dwb). The observed protein content in cricket and amaranth enriched porridge flour is higher than that reported by Agbemafle et al. [46] in complementary food with orange-fleshed sweet potato, enriched with palm weevil larvae. The high protein in our formulated porridge products may be accredited to the addition of cricket and amaranth powder, which are known to be highly rich in protein [47].

The crude protein of the various porridge products fortified with cricket and amaranth meal varied slightly. This variation could be associated with the amino acids being metabolized into ammonia and other volatile flavour compounds during the various process methods, especially during fermentation [48]. A similar trend was reported during the fermentation of pearl millet by Osman [49]. Conversely, there was a slight increase in the protein content of the germinated product, which could be attributed to the mobilization of storage nitrogen and the synthesis of enzymatic proteins by the sprouting seeds during germination [50].

Fat content is an important factor in influencing the energy density of foods. It also provides essential FAs, improves the absorption of fat-soluble vitamins, and the sensory quality of food [51]. The fat content of formulated porridge products with cricket and amaranth meal was higher than that of commercial porridge products. Fermentation process caused a 13% reduction in fat content, indicating a possible active utilization of fats by microorganisms. These findings are in agreement with the observations by Assohoun et al. [52] who reported a reduction in fat content in fermented maize porridge products. The fiber content was increased during germination but reduced during fermentation and roasting. The increase in fibre content during germination could be attributed to the utilization of sugars by spouting seeds [53]. According to the Codex Alumentarius [54], the energy requirement for complementary foods is 400 kcal/100 g on dry weight basis. The energy contents in all the processed and formulated porridge products ranged from 408.12 to 413.92 kcal/100 g, which is higher compared to the commercial porridge flour (381.28 kcal/100 g). This implies that the new porridge products can sufficiently and adequately meet everyone’s recommended energy allowance and nutrient needs.

Fatty acids, especially PUFAs, have enormous health benefits, including preventing degenerative ailments and promoting brain development and proper immune functioning [55,56]. The intake of omega-3 FAs during pregnancy and lactation reduces mortality and allergies in infants and improves their cognitive functions [57,58]. The formulations varied in the composition of FA with the fermented porridge products integrated with cricket and amaranth meal having the highest proportion of PUFAs, including the omega-3 FAs. The ratios of omega-6 to omega-3 FAs were lower than those reported by Paucean et al. [59] on wheat-lentil composite flour and Cheseto et al. [27] on insect oils and cookies. The fermentation process caused an increase in the levels of PUFAs and MUFAs and a decrease in the SFAs. Two omega-3 FAs (ALA + EPA) were detected in all the newly formulated flour products, but DHA was only present in the fermented porridge product, an indication of biosynthesis of DHA during fermentation. The increase in PUFAs, particularly omega-3 FAs, during fermentation could be attributed to microbial synthesis [60]. In this study, roasting increased the amounts of SFAs and oleic acid but decreased the amounts of unsaturated linoleic acid and omega-3 FAs. Studies indicate that the rate of FA oxidation increases as the number of double bonds increases, hence PUFAs will isomerize at a higher rate than MUFAs (oleic acid) [61], which partly explains the increase and decrease of oleic and linoleic acids, respectively, during roasting. Germination caused a 9% decrease in MUFAs and 12% increase in the levels of PUFAs. The resulted in an increase in PUFAs during germination, which is similar to the findings reported by Mariod et al. [62] on germinated black cumin seeds.

The formulated porridge products had increased amounts of essential minerals including zinc and iron. The iron content in the formulated products (8.6–19.5 mg/100 g) met the 7 mg/day recommended daily allowance (RDA) for iron in young children aged 1–3 years. Zinc content was in the range of 3.1–3.7 mg/100 g, which contributes about 75–90% RDA for zinc (4.1 mg/day) in young children. All formulated porridge products were high in calcium content (234.9–312.8 mg/100 g), although the levels were inadequate for providing the RDA (500 mg/day) for all the target age groups [11]. Fermentation processing only reduced the concentrations of Mg and P, while increasing that of Mn. reduction in mineral content during fermentation, has also been observed in previous studies involving cowpea porridge flour [63]. However, germination has been reported to increase the levels of Ca and Fe, which is consistent with previously reported studies by Tizazu et al. [64]. The increase in mineral content could be attributed to losses of water-soluble compounds during soaking.

Vitamins are organic compounds that are required in small amounts for the maintenance of normal health and biological reactions in the cells [65]. In this study, the fermentation process caused an increase in vitamin C, thiamine (B_1_), pyridoxine (B_6_), pantothenic acid (B_5_), folate (B_9_), and nicotinamide. The increase in water-soluble vitamins, particularly of the B-group, during fermentation could be as a result of microbial synthesis [66]. The results are in agreement with Kaprasob et al. [67] who reported an increase in B-group vitamins during fermentation of cashew apples, using probiotic strains of lactic acid bacteria (LAB). However, fermentation reduced nicotinic acid and vitamin B_12_ in the various porridge products, and the losses might be attributed to utilization by LAB in their metabolic biosynthesis necessary for growth [68]. Germination on the other hand, enhanced the concentrations of vitamin C, nicotinic acid, vitamin B_6_, B_5,_ and B_9_. The increase could be due to the synthesis of these vitamins by the germinating seeds [69]. The increase in vitamin C during germination has been reported by other researchers and is attributed to the enzymatic hydrolysis of starch by amylases, leading to an increase in the bioavailability of glucose for biosynthesis of vitamin C [69,70]. However, the loss of thiamine to undetectable levels was recorded in the germinated porridge products; this might be due to leaching in the spouting medium [71]. The slight effect of roasting on riboflavin and nicotinamide in porridge flours suggests high thermal stability of B group vitamins [72]. The germination process increased the α-tocopherol content, and these results are comparable to those reported by Young et al. [73] on germinated rough rice seeds. The roasting process, however, did not affect the levels of tocopherols; this could be due to the fact that tocopherols are resistant to thermal degradation [74]. Contrarily, Stuetz et al. [75] reported the loss of tocopherols in roasted nuts compared to raw nuts; this variation might be explainable by the different roasting conditions used in the study.

Phytic acid is the principal storage form of phosphorous in plant seeds [76] and is known to reduce the bioavailability of dietary Zn, Fe, and Ca in humans and monogastric animals, owing to their chelating properties [77]. Both fermented and germinated porridge products had reduced levels of phytic acid when compared to the unprocessed sample. The reduction in phytic acid in the fermented product may be attributed to the effect of microbial phytase and the low pH which favours the activity of endogenous cereal phytase during fermentation [78]. Phytic acid degradation during the germination process could be due to increased activity of enzyme phytase in spouting grains which hydrolyse phytic acid into lower inositol phosphates [79]. Germination decreased phytic acid by 67% as compared to fermentation at 33%. The effect of germination on phytic acid levels could be attributed to a combination of processes such as phytase activity and the leaching of phytate ions into water during soaking before the germination of grains. Minor degradation of phytic acid during fermentation could also be attributed to the high phytic acid content and presence of endogenous phytase inhibitory compounds, such as tannins in both finger millet and amaranth grains [80]. Fermentation is more effective when carried out in grains with low anti-nutrients levels [81]. Contrarily, the increase in phytic acid during roasting could be as a result of an increase in lower phosphorylated inositol phosphates, which in turn increased the total phytic acid content. Similar results were observed by Frontela et al. [82]. However, our study did not differentiate between the classes of phytates but rather focused on the total phytic acid content. Moreover, the roasting temperature (120 °C) restricted endogenous phytase enzyme activity whose optimum temperature varies from 35 to 80 °C [83]. The low levels of phytic acid in the commercial porridge flour could be due to the use of exogenous phytate degrading enzymes during the industrial processing of the flour. The complete degradation of phytic acid in the four formulated porridge products is feasible with the use of additional exogenous phytases [83].

The phytate/mineral molar ratio is an indicator of mineral bioavailability in plant–based foods [33]. Flour samples from germinated grains had good bioavailability of Zn, while all porridge products, except for the roasted sample, had good bioavailability of Ca. The increased bioavailability of minerals corresponds with the reduction in phytic acid. The commercial porridge flour had good bioavailability of minerals except for Fe, and this could be attributed to low levels of phytic acid as well as the addition of extra minerals during fortification (as stated in the label).

Flavonoids exhibit powerful antioxidative properties, which makes them significant in the prevention of degenerative diseases [84]. The total flavonoid contents in our products ranged between 59.8–165.5 mg/100 g. Flavonoid content increased during fermentation, and this is consistent with the observations by Adetuyi and Ibrahim [85], who reported an increase in total flavonoid content during the fermentation of okra seeds. The increase in flavonoid content could be attributed to the release of simple phenolic compounds during the acid and microbial hydrolysis of complex phenolic compounds during fermentation [86]. A reduction in flavonoid content was noted during germination (42%) and roasting (10%). The reduction during roasting could be due to flavonoids’ sensitivity to high temperatures [87]. The results on the reduction of flavonoids during roasting are similar to the findings of other authors [88].

Tannins are regarded as antinutrients because of their ability to inhibit digestive enzymes, lowering the digestibility of most nutrients, particularly proteins [89]. Tannin content reduced during germination is probably due to the complexing of tannins with seed proteins and metabolic enzymes [90] and leaching into the sprouting medium [91]. Significant increase in tannin content observed during fermentation might be due to the hydrolysis of various components, including condensed tannins by catabolic enzymes. Similar findings were reported by Osman [49] during the fermentation of pearl millet. However, reductions in tannin content during fermentation have also been reported [92].

## 5. Conclusions

The projected growth in global population with the attendant increase in food demands, especially in the African region, calls for focusing and devoting more attention to the underutilized crop and animal species, which have great potential to influence and improve food security for African nations and promote sustainable rural growth and development. Grain amaranth and edible insects are some of such neglected and underutilized species, despite their great inherent health-promoting components, which are good for human applications and uses. The demand for indigenous vegetable/grains and cricket-based products have risen dramatically in recent years because of the rapidly expanding human population. Our findings have also demonstrated the importance of integrating cricket and amaranth grain meal into porridge products to produce nutritious food sufficient for meeting the recommended daily allowance of the most vulnerable segments of the population, if processed properly. The comparative analysis of various processing protocols would empower communities to develop acceptable composite porridge products that are appealing to the consumers and increase demand. The four porridge products developed in this study are higher in energy density and nutritional value, as evidenced by the improved fat, protein, vitamin, and mineral contents in the various products, compared to the widely consumed commercial porridge product. Although complete degradation of phytic acid was not achieved, this study demonstrated that germination and fermentation techniques played a key role in improving the bioavailability of nutrients in the porridge products. Additional, innovative traditional processing technologies to increase phytic acid degradation are warranted. From a nutritional point of view, further studies on protein digestibility and utilization, the bioavailability of essential mineral and trace elements as well as other factors such as pH, the concentration of enhancers, and inhibitors (dietary fibre and polyphenols) would be crucial, particularly for cricket-based food products.

## Figures and Tables

**Table 1:** Proximate and energy values of porridge flour on a dry weight basis.

Products	Proximate Composition						
	Moisture (%)	Ash (g/100 g)	Fiber (g/100 g)	Protein (g/100 g)	Fat (g/100 g)	CHO (g/100 g)	Energy (kcal/100 g)
CPF	11.76 ± 0.25 ^e^	2.57 ± 0.04 ^ab^	4.79 ± 0.28 ^c^	8.55 ± 0.16 ^a^	2.14 ± 0.16 ^a^	81.94 ± 0.26 ^d^	381.28 ± 0.35 ^a^
FFM–AC	4.84 ± 0.25 ^b^	2.22 ± 0.15 ^a^	3.33 ± 0.29 ^a^	15.34 ± 0.17 ^b^	7.22 ± 0.06 ^b^	71.88 ± 0.29 ^c^	413.92 ± 0.80 ^c^
GFM–AC	5.92 ± 0.42 ^c^	2.88 ± 0.48 ^b^	5.34 ± 0.02 ^d^	16.12 ± 0.15 ^d^	8.19 ± 0.09 ^c^	67.46 ± 0.39 ^a^	408.12 ± 2.27 ^b^
RFM–AC	3.02 ± 0.22 ^a^	2.76 ± 0.05 ^ab^	3.88 ± 0.04 ^b^	15.54 ± 0.16 ^bc^	8.08 ± 0.28 ^c^	69.74 ± 0.13 ^b^	413.83 ± 1.44 ^c^
UFM–AC	7.44 ± 0.26 ^d^	2.83 ± 0.09 ^ab^	4.64 ± 0.11 ^c^	15.90 ± 0.28 ^cd^	8.31 ± 0.38 ^c^	68.31 ± 0.71 ^a^	411.68 ± 1.71 ^bc^

Products: CPF = commercial porridge flour; FFM–AC = fermented finger millet − amaranth + cricket; GFM–AC = germinated finger millet − amaranth + cricket; RFM–AC = roasted finger millet − amaranth + cricket; UFM–AC = unprocessed finger millet − amaranth + cricket. Values are mean (± standard deviation). Moisture content not based on dry weight. Mean values with different superscript letters within columns are significantly different at *p* < 0.05 according to the Tukey test.

**Table 2:** Compositions of fatty acids (µg/g of oil) of porridge flour samples analysed using gas chromatography coupled with mass spectrometry (GC-MS).

Peak No.	tR (min)	Compound Name	ω-n(Δn)	CPF	FFM-AC	GFM-AC	RFM-AC	UFM-AC
1	18.96	Methyl Dodecanoate	C12:0	-	0.67 ± 0.09	0.27 ± 0.01	0.58 ± 0.05	0.35 ± 0.02
2	19.72	Methyl 11-Methyldodecanoate	Iso-methyl-C12:0	-	0.05 ± 0.01	0.06 ± 0.00	-	
3	20.12	Methyl Tridecanoate	C13:0	-	0.12 ± 0.01	0.06 ± 0.00	0.09 ± 0.01	0.66 ± 0.00
4	20.82	Methyl 12-Methyltridecanoate	Iso-methyl-C13:0	-	0.21 ± 0.02	0.12 ± 0.00	0.07 ± 0.01	0.13 ± 0.01
5	21.22	Methyl Tetradecanoate	C14:0	0.85 ± 0.04	14.72 ± 0.84	6.09 ± 0.10	12.04 ± 1.21	8.26 ± 0.97
6	21.78	Methyl 4-Methyldodecanoate	Iso-methyl-C12:0	-	0.72 ± 0.07	0.44 ± 0.00	-	-
7	22.00	Methyl 13-Methyltetradecanoate	Iso-methyl-C14:0	-	3.64 ± 0.21	1.98 ± 0.01	1.42 ± 0.17	0.89 ± 0.17
8	22.00	Methyl 12-Methyltetradecanoate	Iso-methyl-C14:0	-	0.85 ± 0.04	0.54 ± 0.00	1.84 ± 0.10	0.29 ± 0.19
9	22.29	Methyl Pentadecanoate	C15:0	0.38 ± 0.04	3.79 ± 0.28	1.61 ± 0.00	3.20 ± 0.48	2.93 ± 0.56
10	22.74	Methyl 5,9,13-Trimethyltetradecanoate	Iso-trimethyl-C14:0	-	0.45 ± 0.05	0.00 ± 0.00	-	-
11	22.94	Methyl 14-Methylpentadecanoate	Iso-methyl-C15:0	-	1.22 ± 0.10	0.85 ± 0.01	1.24 ± 0.07	0.94 ± 0.06
12	23.37	Methyl Hexadecanoate	C16:0	32.22 ± 1.37	200.94 ± 6.55	255.32 ± 7.40	559.63 ± 50.55	319.22 ± 28.46
13	23.93	Methyl 15-Methylhexadecanoate	Iso-methyl-C16:0	-	3.89 ± 0.38	2.90 ± 0.06	3.09 ± 0.42	3.15 ± 0.39
14	24.02	Methyl 14-Methylhexadecanoate	Iso-methyl-C16:0	1.30 ± 0.13	11.82 ± 0.94	5.85 ± 0.12	12.80 ± 1.11	6.23 ± 0.88
15	24.29	Methyl Heptadecanoate	C17:0	1.11 ± 0.22	9.92 ± 0.71	3.08 ± 0.05	7.40 ± 0.68	4.58 ± 0.91
16	24.69	Methyl 14-Methylheptadecanoate	Iso-methyl-C17:0	-	1.40 ± 0.18	1.43 ± 0.02	2.99 ± 0.24	1.66 ± 0.20
17	25.25	Methyl Octadecanoate	C18:0	13.02 ± 0.51	170.54 ± 2.98	78.14 ± 1.41	36.71 ± 2.68	114.00 ± 23.09
18	26.12	Methyl Nonadecanoate	C19:0	-	2.08 ± 0.14	1.46 ± 0.05	1.92 ± 0.06	1.44 ± 0.30
19	26.98	Methyl Eicosanoate	C20:0	3.05 ± 0.06	13.07 ± 0.15	11.07 ± 0.00	26.99 ± 1.49	16.48 ± 2.30
20	27.58	Methyl 18-Methyleicosanoate	Iso-methyl-C20:0	-	4.90 ± 0.78	3.45 ± 0.00	5.11 ± 0.76	2.80 ± 0.17
21	27.80	Methyl Heneicosanoate	C21:0	-	2.30 ± 0.23	3.34 ± 0.30	3.75 ± 0.66	1.79 ± 0.10
22	28.59	Methyl Docosanoate	C22:0	2.47 ± 0.02	5.35 ± 0.13	6.30 ± 0.13	9.01 ± 0.45	6.92 ± 0.85
23	29.37	Methyl Tricosanoate	C23:0	-	3.95 ± 0.09	3.93 ± 0.01	4.63 ± 0.39	2.89 ± 0.21
24	30.13	Methyl Tetracosanoate	C24:0	-	9.45 ± 0.54	6.07 ± 0.04	10.98 ± 0.48	4.43 ± 1.23
		∑ SFA						
25	20.95	Methyl 11Z-Tetradecenoate	C14:1 (n-3)	-	0.52 ± 0.01	-	0.93 ± 0.09	-
26	21.08	Methyl 9Z-Tetradecenoate	C14:1 (n-3)	-	0.89 ± 0.09	-	0.67 ± 0.07	-
27	23.12	Methyl 9Z-Hexadecenoate	C16:1 (n-7)	2.24 ± 0.26	55.80 ± 1.46	18.50 ± 0.16	67.70 ± 1.84	31.80 ± 2.43
28	24.09	Methyl 10Z-Heptadecenoate	C17:1 (n-7)	-	7.42 ± 1.10	1.37 ± 0.03	1.75 ± 0.16	0.81 ± 0.08
29	25.00	Methyl 11-Octadecenoate	C18:1 (n-9)	-	3.69 ± 0.18	3.48 ± 0.11	2.83 ± 0.19	3.72 ± 0.12
30	25.07	Methyl 9E-Octadecenoate	C18:1 (n-9)	111.68 ± 6.88	729.29 ± 41.54	630.70 ± 12.89	1149.35 ± 93.82	332.62 ± 40.54
31	25.88	Methyl 10-Nonadecenoate	C19:1 (n-9)	-	2.69 ± 0.14	3.81 ± 0.17	8.99 ± 0.45	4.55 ± 0.46
32	26.77	Methyl 11Z-Eicosenoate	C20:1 (n-9)	2.94 ± 0.14	8.63 ± 0.55	8.52 ± 0.16	19.96 ± 0.57	11.31 ± 2.20
33	28.41	Methyl 11-Docosenoate	C22:1 (n-11)	-	4.23 ± 0.20	-	3.27 ± 0.30	-
34	29.95	Methyl 15Z-Tetracosenoate	C24:1 (n-9)	-	3.31 ± 0.16	-	-	-
		∑ MUFA						
35	24.74	Methyl 9Z,12Z-Octadecadienoate	C18:2 (n-6)	-	74.70 ± 4.02	38.38 ± 2.09	16.69 ± 1.10	22.62 ± 2.07
36	24.76	methyl 6Z,9Z,12Z-Octadecatrienoate	C18:3 (n-3)	-	4.60 ± 0.43	3.48 ± 0.40	1.67 ± 0.15	2.80 ± 0.03
37	25.41	Methyl 7,12-Octadecadienoate	C18:2 (n-7)	-	8.87 ± 0.66	11.07 ± 0.94	6.55 ± 1.96	5.22 ± 0.27
38	25.79	Methyl 9Z,11E-Octadecadienoate	C18:2 (n-7)	-	2.39 ± 0.09	2.60 ± 0.42	1.62 ± 0.21	1.35 ± 0.01
39	26.24	Methyl 9Z,11E,13E-Octadecatrienoate (α-ESA)	C18:3 (n-3)	-	1.03 ± 0.04	0.92 ± 0.10	0.57 ± 0.00	0.74 ± 0.03
40	26.26	Methyl 9Z,12Z,15Z-Octadecatrienoate (ALA)	C18:3 (n-3)	-	7.01 ± 0.23	7.10 ± 0.61	3.79 ± 0.01	5.07 ± 0.02
41	26.44	Methyl 5Z,8Z,11Z,14Z-Eicosatetraenoate (AA)	C20:4 (n-6)	-	1.55 ± 0.09	2.76 ± 0.06	1.89 ± 0.04	1.95 ± 0.19
42	26.50	Methyl 5Z,8Z,11Z,14Z,17Z-Eicosapentaenoate (EPA)	C20:5 (n-3)	-	13.07 ± 0.77	4.44 ± 0.02	-	-
43	26.64	Methyl 8,11,14,17-Eicosatetraenoate (AA)	C20:4 (n-6)	-	2.040 ± 0.10	-	-	-
44	28.07	Methyl 4Z,7Z,10Z,13Z,16Z,19Z-Docosahexaenoate (DHA)	C22:6 (n-3)	-	2.40 ± 0.34	-	-	-
		∑ PUFA		-	117.66 ± 3.17	70.76 ± 1.30	32.68 ± 1.72	39.73 ± 2.16
		∑ n-6 PUFA		-	89.55 ± 4.42	54.82 ± 0.92	26.65 ± 1.68	31.13 ± 2.15
		∑ n-3 PUFA		-	28.11 ± 0.75	15.94 ± 0.99	6.03 ± 1.15	8.60 ± 0.01
		∑ n-6/n-3		-	3.2	3.4	4.4	3.6
		∑ ALA + EPA + DHA		-	27.08 ± 0.70	15.02 ± 1.00	5.47 ± 1.15	7.87 ± 0.04

(tR Retention time, Mean ± standard deviation). SFA = saturated fatty acids, MUFA = monounsaturated fatty acids, PUFA = polyunsaturated fatty acids, ALA = α -Linolenic acid, EPA = Eicosapentaenoic acid, DHA = Docosapentaenoic acid, α-ESA = alpha Eleostearic acid.

**Table 3:** Vitamin content (mg/100 g) in porridge flour products.

Vitamins	Porridge Products				
	CPF	FFM–AC	GFM–AC	RFM–AC	UFM–AC
Vitamin C	149.6 ± 2.1 ^c^	146.5 ± 2.8 ^c^	72.0 ± 6.5 ^b^	55.2 ± 2.9 ^a^	58.0 ± 5.1 ^a^
Thiamine (B_1_)	–	39.5 ± 3.0 ^c^	–	4.3 ± 0.2 ^b^	5.9 ± 0.4 ^b^
Nicotinic acid (B_3_)	27.7 ± 2.9 ^e^	–	19.5 ± 1.2 ^d^	6.1 ± 0.5 ^b^	10.6 ± 0.7 ^c^
Pyridoxine (B_6_)	0.5 ± 0.1 ^a^	10.8 ± 1.1 ^c^	6.0 ± 0.3 ^b^	–	–
Nicotinamide	3.0 ± 0.3 ^a^	47.9 ± 2.1 ^d^	33.9 ± 1.1 ^c^	7.1 ± 0.2 ^b^	8.8 ± 0.4 ^b^
Pantothenic acid (B_5_)	26.4 ± 2.5 ^a^	423.3 ± 3.4 ^d^	453.8 ± 44.5 ^d^	314.4 ± 18.4 ^c^	209.6 ± 3.8 ^b^
Folate (B_9_)	28.6 ± 2.6 ^a^	38.8 ± 1.5 ^b^	42.4 ± 3.7 ^b^	41.8 ± 0.3 ^b^	29.3 ± 1.8 ^a^
Cyanocobalamin (B_12_)	3.2 ± 0.3 ^a^	21.9 ± 1.4 ^c^	13.7 ± 4.0 ^b^	12.4 ± 0.9 ^b^	37.7 ± 4.0 ^d^
Riboflavin (B_2_)	74.2 ± 8.2 ^b^	41.6 ± 1.0 ^a^	34.8 ± 2.9 ^a^	45.5 ± 2.4 ^a^	41.6 ± 5.0 ^b^
Retinol	0.55 ± 0.03 ^c^	0.54 ± 0.08 ^c^	0.07 ± 0.01 ^a^	0.29 ± 0.01 ^ab^	0.38 ± 0.19 ^bc^
γ-Tocopherols	0.88 ± 0.04 ^c^	0.17 ± 0.01 ^a^	0.19 ± 0.01 ^a^	0.54 ±0.00 ^b^	0.52 ± 0.02 ^b^
α-Tocopherols	0.46 ± 0.06 ^a^	0.35 ± 0.09 ^a^	1.48 ± 0.03 ^c^	0.76 ± 0.08 ^b^	0.83 ± 0.03 ^b^

Porridge product: FFM–AC = fermented finger millet − amaranth + cricket; RFM–AC = roasted finger millet − amaranth + cricket; GFM–AC = germinated finger millet − amaranth + cricket; UFM–AC = unprocessed finger millet − amaranth + cricket; CPF = commercial porridge flour. Values are mean (± standard deviation). Mean values with different superscript letters within rows are significantly different at *p* < 0.05, according to the Tukey test.

**Table 4:** Mineral content and bioavailability of porridge flour formulations.

Minerals	Porridge Products				
	CPF	FFM–AC	GFM–AC	RFM–AC	UFM–AC
Mg (mg/100 g)	145.08 ± 0.25 ^a^	169.57 ± 8.03 ^b^	207.82 ± 11.93 ^c^	210.84 ± 1.45 ^c^	203.09 ± 5.14 ^c^
Fe (mg/100 g)	9.86 ± 2.08 ^a^	8.56 ± 1.45 ^a^	19.48 ± 6.69 ^b^	9.18 ± 1.18 ^a^	9.55 ± 2.42 ^a^
Ca (mg/100 g)	312.69 ± 0.57 ^c^	234.87 ± 17.60 ^a^	278.61 ± 17.90 ^b^	257.69 ± 1.37 ^ab^	244.69 ± 4.94 ^a^
Zn (mg/100 g)	1.86 ± 0.04 ^a^	3.23 ± 0.28 ^b^	3.71 ± 0.18 ^b^	3.39 ± 0.31 ^b^	3.08 ± 0.16 ^b^
P (mg/100 g)	221.63 ± 5.57 ^a^	372.71 ± 19.14 ^b^	469.28 ± 9.55 ^c^	458.70 ± 3.76 ^c^	476.72 ± 17.46 ^c^
Mn (mg/100 g)	22.44 ± 0.20 ^c^	10.92 ± 0.63 ^b^	9.32 ± 0.67 ^a^	9.45 ± 0.15 ^a^	8.64 ± 0.04 ^a^
Cu (µg/100 g)	477.42 ± 1.88 ^a^	728.78 ± 15.37 ^b^	787.20 ± 42.28 ^b^	724.94 ± 22.80 ^b^	736.28 ± 14.47 ^b^
Molar ratios (Bioavailability)					
Phy:Fe	1.46 ± 0.05 ^a^	5.69 ± 1.38 ^b^	1.31 ± 0.42 ^a^	9.63 ± 1.41 ^c^	7.76 ± 1.90 ^bc^
Phy:Zn	9.03 ± 1.97 ^a^	17.27 ± 1.40 ^b^	7.43 ± 0.64 ^a^	30.48 ± 5.39 ^c^	26.90 ± 2.04 ^c^
Phy:Ca	0.03 ± 0.01 ^a^	0.14 ± 0.00 ^c^	0.06 ± 0.01 ^a^	0.24 ± 0.01 ^e^	0.21 ± 0.00 ^d^

Porridge flour product: FFM–AC = fermented finger millet − amaranth + cricket; RFM–AC = roasted finger millet − amaranth + cricket; GFM–AC = germinated finger millet − amaranth + cricket meal; UFM–AC = unprocessed finger millet − amaranth + cricket; CPF = commercial porridge flour. Values are mean (± standard deviation). Mean values with different superscripts within rows are significantly different at *p* < 0.05 according to the Tukey test.

## Data Availability

Data are contained within the article.

## References

[1] Wanjala G., Onyango A., Makayoto M., Onyango C. (2016). Indigenous Technical Knowledge and Formulations of Thick (Ugali) and Thin (Uji) Porridges Consumed in Kenya. Afr. J. Food Sci..

[2] Békés F., Schoenlechner R., Tömösközi S., Wrigley C. (2017). Ancient wheats and pseudocereals for possible use in cereal-grain dietary intolerances. Cereal Grains.

[3] Liceaga A.M. (2021). Processing insects for use in the food and feed industry. Curr. Opin. Insect. Sci..

[4] Nyangena D.N., Mutungi C., Imathiu S., Kinyuru J., Affognon H., Ekesi S., Nakimbugwe D., Fiaboe K.K. (2020). Effects of Traditional Processing Techniques on the Nutritional and Microbiological Quality of Four Edible Insect Species Used for Food and Feed in East Africa. Foods.

[5] Fombong F.T., Van Der Borght M., Vanden Broeck J. (2017). Influence of Freeze-Drying and Oven-Drying Post Blanching on the Nutrient Composition of the Edible Insect *Ruspolia Differens*. Insects.

[6] Dobermann D., Field L.M., Michaelson L.V. (2019). Impact of Heat Processing on the Nutritional Content of Gryllus Bimaculatus (Black Cricket). Nutr. Bull..

[7] Mutungi C., Irungu F.G., Nduko J., Mutua F., Affognon H., Nakimbugwe D., Ekesi S., Fiaboe K.K.M. (2019). Postharvest Processes of Edible Insects in Africa: A Review of Processing Methods, and the Implications for Nutrition, Safety and New Products Development. Crit. Rev. Food Sci. Nutr..

[8] Ssepuuya G., Nakimbugwe D., De Winne A., Smets R., Claes J., Van Der Borght M. (2020). Effect of Heat Processing on the Nutrient Composition, Colour, and Volatile Odour Compounds of the Long-Horned Grasshopper *Ruspolia differens* Serville. Food Res. Int..

[9] Mmari M.W., Kinyuru J.N., Laswai H.S., Okoth J.K. (2017). Traditions, Beliefs and Indigenous Technologies in Connection with the Edible Longhorn Grasshopper *Ruspolia differens* (Serville 1838) in Tanzania. J. Ethnobiol. Ethnomed..

[10] Onyango C., Okoth M.W., Mbugua S.K. (2000). Effect of Drying Lactic Fermented *Uji* (an East African Sour Porridge) on Some Carboxylic Acids. J. Sci. Food Agric..

[11] WHO, FAO (2004). WHO|Vitamin and Mineral Requirements in Human Nutrition. World Health Organization WHO/FAO (2004).

[12] WHO, FAO, UNU (2007). Joint FAO/WHO/UNU Expert Consultation on Protein and Amino Acid Requirements in Human Nutrition (2002: Geneva, Switzerland).

[13] Latimer G.W.J., AOAC (2019). Official Methods of Analysis of AOAC International.

[14] Ochieng J., Schreinemachers P., Ogada M., Dinssa F.F., Barnos W., Mndiga H. (2019). Adoption of improved amaranth varieties and good agricultural practices in East Africa. Land Use Policy.

[15] Yang R.Y., Keding G.B. (2009). Nutritional Contributions of Important African Indigenous Vegetables. African Indigenous Vegetables in Urban Agriculture.

[16] Petr J., Michalik I., Tlaskalova H., Capouchova I., Famera O., Urminska D., Tukova L., Knoblochova H. (2003). Extension of the spectra of plant products for the diet in celiac disease. Czech J. Food Sci..

[17] Macharia-Mutie C.W., van de Wiel A.M., Moreno-Londono A.M., Mwangi A.M., Brouwer I.D. (2011). Sensory acceptability and factors predicting the consumption of grain amaranth in Kenya. Ecol. Food Nutr..

[18] Karamać M., Gai F., Longato E., Meineri G., Janiak M.A., Amarowicz R., Peiretti P.G. (2019). Antioxidant Activity and Phenolic Composition of Amaranth (*Amaranthus caudatus*) during Plant Growth. Antioxidants.

[19] Chavez-Jauregui R.N., Silva M.E.M.P., Arěas J.A.G. (2000). Extrusion cooking process for amaranth. J. Food Sci..

[20] Olaniyi J.O. (2007). Evaluation of yield and quality performance of grain amaranth varieties in the southwestern Nigeria. Res. J. Agron..

[21] Kolawole E.L., Sarah O.A. (2009). Growth and yield performance of Amaranthus cruentus influenced by planting density and poultry manure application. Not. Bot. Horti Agrobot. Cluj-Napoca..

[22] Kim H.K., Kim M.J., Cho H.Y., Kim E.-K., Shin D.H. (2006). Antioxidative and anti-diabetic effects of amaranth (*Amaranthus esculantus*) in streptozotocin-induced diabetic rats. Cell Biochem. Funct..

[23] Kim H.K., Kim M.J., Shin D.H. (2006). Improvement of lipid profile by amaranth (*Amaranthus esculantus*) supplementation in streptozotocin-induced diabetic rats. Ann. Nutr. Metab..

[24] Bario D.A., Añón M.C. (2010). Potential antitumor properties of a protein isolate obtained from the seeds of *Amaranthus mantegazzianus*. Eur. J. Nutr..

[25] Aderibigbe O.R., Ezekiel O.O., Owolade S.O., Korese J.K., Sturm B., Hensel O. (2022). Exploring the potentials of underutilized grain amaranth (*Amaranthus* spp.) along the value chain for food and nutrition security: A review. Crit. Rev. Food Sci. Nutr..

[26] Okoth J.K., Ochola S., Gikonyo N.K., and Makokha A.O. (2017). Efficacy of amaranth sorghum grains porridge in rehabilitating moderately acute malnourished children in a low-resource setting in Kenya: A randomized controlled trial. Integr. Food Nutr. Metal..

[27] Cheseto X., Baleba S.B.S., Tanga C.M., Kelemu S., Torto B. (2020). Chemistry and Sensory Characterization of a Bakery Product Prepared with Oils from African Edible Insects. Foods.

[28] Thermo Fisher Scientific (2010). Determination of Water–and Fat–Soluble Vitamins by HPLC, Knowledge Creation Diffusion Utilization.

[29] Bhatnagar-Panwar M., Bhatnagar-Mathur P., VijayAnand Bhaaskarla V., Reddy Dumbala S., Sharma K.K. (2015). Rapid, Accurate and Routine HPLC Method for Large-Scale Screening of pro-Vitamin A Carotenoids in Oilseeds. J. Plant Biochem. Biotechnol..

[30] Megazyme (2017). Megazyme—Phytic Acid (Phytate)/Total Phosphoru Assay Kit Procedure.

[31] Saxena V., Mishra G., Saxena A., Vishwakarma K.K. (2013). A Comparative Study on Quantitative Estimation of Tannins in Terminalia Chebula, Terminalia Belerica, Terminalia Arjuna and Saraca Indica Using Spectrophotometer. Asian J. Pharm. Clin. Res..

[32] Zhishen J., Mengcheng T., Jianming W. (1999). The Determination of Flavonoid Contents in Mulberry and Their Scavenging Effects on Superoxide Radicals. Food Chem..

[33] Norhaizan M.E., Nor Faizadatul Ain A.W. (2009). Determination of Phytate, Iron, Zinc, Calcium Contents and Their Molar Ratios in Commonly Consumed Raw and Prepared Food in Malaysia. Malays. J. Nutr..

[34] R Core Team (2020). R: A Language and Environment for Statistical Computing. R Foundation for Statistical Computing.

[35] Anigo K., Ameh D., Ibrahim S., Danbauchi S. (2010). Nutrient Composition of Complementary Food Gruels Formulated from Malted Cereals, Soybeans and Groundnut for Use in North-Western Nigeria. Afr. J. Food Sci..

[36] Dewey K.G. (2013). The Challenge of Meeting Nutrient Needs of Infants and Young Children during the Period of Complementary Feeding: An Evolutionary Perspective. J. Nutr..

[37] Murugu D.K., Onyango A.N., Ndiritu A.K., Osuga I.M., Xavier C., Nakimbugwe D., Tanga C.M. (2021). From Farm to Fork: Crickets as Alternative Source of Protein, Minerals, and Vitamins. Front. Nutr..

[38] Melgar-Lalanne G., Hern’andez-´Alvarez A., Salinas-Castro A. (2019). Edible insects processing: Traditional and innovative technologies. Compr. Rev. Food Sci. Food Saf..

[39] Lillioja S., Neal A.L., Tapsell L., Jacobs D.R. (2013). Whole grains, type 2 diabetes, coronary heart disease, and hypertension: Links to the aleurone preferred over indigestible fiber. BioFactors.

[40] Onyango C.A., Ochanda S.O., Mwasaru M.A., Ochieng J.K., Mathooko F.M., Kinyuru J.N. (2013). Effects of Malting and Fermentation on Anti-Nutrient Reduction and Protein Digestibility of Red Sorghum, White Sorghum and Pearl Millet. J. Food Res..

[41] Min Z., Chen H., Li J., Pei Y., Liang Y. (2010). Antioxidant properties of tartary buckwheat extracts as affected by different thermal processing methods. LWT Food Sci. Technol..

[42] Masarirambi M., Mavuso V., Songwe V., Nkambule T., and Mhazo N. (2010). Indigenous Post-Harvest Handling and Processing of Traditional Vegetables in Swaziland: A Review. Afr. J. Agric. Res..

[43] Folch J., Lees M., Sloane Stanley G.H. (1957). A Simple Method for the Isolation and Purification of Total Lipides from Animal Tissues. J. Biol. Chem..

[44] Luchuo E.B., Paschal K.A., Geraldine N., Kindong N.P., Nsah Y.S., Tanjeko A.T. (2013). Malnutrition in Sub—Saharan Africa: Burden, causes and prospects. Pan Afr Med. J..

[45] Branca F., Demaio A., Udomkesmalee E., Baker P., Aguayo V.M., Barquera S., Dain K., Keir L., Lartey A., Mugambi G. (2020). Dynamics of the double burden of malnutrition and the changing nutrition reality. Lancet.

[46] Agbemafle I., Hadz D., Amagloh F.K., Zotor F.B., Reddy M.B. (2020). Orange-Fleshed Sweet Potato and Edible Insects. Foods.

[47] Magara H.J.O., Niassy S., Ayieko M.A., Mukundamago M., Egonyu J.P., Tanga C.M., Kimathi E.K., Ongere J.O., Fiaboe K.K.M., Hugel S. (2021). Edible Crickets (Orthoptera) Around the World: Distribution, Nutritional Value, and Other Benefits—A Review. Front. Nutr..

[48] Pranoto Y., Anggrahini S., Efendi Z. (2013). Effect of Natural and Lactobacillus Plantarum Fermentation on In-Vitro Protein and Starch Digestibilities of Sorghum Flour. Food Biosci..

[49] Osman M.A. (2011). Effect of Traditional Fermentation Process on the Nutrient and Antinutrient Contents of Pearl Millet during Preparation of Lohoh. J. Saudi Soc. Agric. Sci..

[50] Nnam N.M. (2000). Chemical Evaluation of Multimixes Formulated from Some Local Staples for Use as Complementary Foods in Nigeria. Plant Foods Hum. Nutr..

[51] Onabanjo O.O., Oguntona C.R.B., Maziya-Dixon B., Olayiwola I.O., Oguntona E.B., Dixon A.G.O. (2008). Nutritional Evaluation of Four Optimized Cassava-Based Complementary Foods. Afr. J. Food Sci..

[52] Assohoun M.C.N., Djeni T.N., Koussémon-Camara M., Brou K. (2013). Effect of Fermentation Process on Nutritional Composition and Aflatoxins Concentration of Doklu, a Fermented Maize Based Food. Food Nutr. Sci..

[53] Ikenebomah M., Kok J.R., Ingram J. (2019). Processing and Fermentation of the African Locust Bean (Parkia Felocoidea) Can. Int. J. Food Sci. Technol..

[54] Codex Alimentarius (1991). Guidelines for Development of Supplementary Foods for Older Infants and Children.

[55] Islam M.S., Castellucci C., Fiorini R., Greco S., Gagliardi R., Zannotti A., Giannubilo S.R., Ciavattini A., Frega N.G., Pacetti D. (2018). Omega-3 Fatty Acids Modulate the Lipid Profile, Membrane Architecture, and Gene Expression of Leiomyoma Cells. J. Cell. Physiol..

[56] Martínez Andrade K.A., Lauritano C., Romano G., Ianora A. (2018). Marine Microalgae with Anti-Cancer Properties. Mar. Drugs.

[57] Stark K.D., Van Elswyk M.E., Higgins M.R., Weatherford C.A., Salem N. (2016). Global Survey of the Omega-3 Fatty Acids, Docosahexaenoic Acid and Eicosapentaenoic Acid in the Blood Stream of Healthy Adults. Prog. Lipid Res..

[58] Gunaratne A.W., Makrides M., Collins C.T. (2012). Maternal Prenatal and/or Postnatal n-3 Fish Oil Supplementation for Preventing Allergies in Early Childhood. Cochrane Database Syst. Rev..

[59] Paucean A., Moldovan O.P., Mureșan V., Socaci S.A., Dulf F.V., Alexa E., Man S.M., Mureșan A.E., Muste S. (2018). Folic Acid, Minerals, Amino-Acids, Fatty Acids and Volatile Compounds of Green and Red Lentils. Folic Acid Content Optimization in Wheat-Lentils Composite Flours. Chem. Cent. J..

[60] Kannan N., Rao A.S., Nair A. (2021). Microbial Production of Omega-3 Fatty Acids: An Overview. J. Appl. Microbiol..

[61] Yoshida H., Abe S., Hirakawa Y., Takagi S. (2001). Roasting Effects on Fatty Acid Distributions of Triacylglycerols and Phospholipids in Sesame (Sesamum Indicum) Seeds. J. Sci. Food Agric..

[62] Mariod A.A., Edris Y.A., Cheng S.F., Abdelwahab S.I. (2012). Effect of Germination Periods and Conditions on Chemical Composition, Fatty Acids and Amino Acids of Two Black Cumin Seeds. Acta Sci. Pol. Technol. Aliment..

[63] Difo H.V., Onyike E., Ameh D.A., Ndidi U.S., Njoku G.C. (2014). Chemical Changes during Open and Controlled Fermentation of Cowpea (Vigna Unguiculata) Flour. Int. J. Food Nutr. Saf..

[64] Tizazu S., Urga K., Abuye C., Retta N. (2010). Improvement of Energy and Nutrient Density of Sorghumbased Complementary Foods Using Germination. Afr. J. Food Agric. Nutr. Dev..

[65] Walther B., Schmid A. (2017). Effect of Fermentation on Vitamin Content in Food.

[66] Barrios-González J. (2012). Solid-State Fermentation: Physiology of Solid Medium, Its Molecular Basis and Applications. Process Biochem..

[67] Kaprasob R., Kerdchoechuen O., Laohakunjit N., Somboonpanyakul P. (2018). B Vitamins and Prebiotic Fructooligosaccharides of Cashew Apple Fermented with Probiotic Strains Lactobacillus Spp., Leuconostoc Mesenteroides and Bifidobacterium Longum. Process Biochem..

[68] Tabaszewska M., Gabor A., Jaworska G., Drożdż I. (2018). Effect of Fermentation and Storage on the Nutritional Value and Contents of Biologically-Active Compounds in Lacto-Fermented White Asparagus (*Asparagus Officinalis* L.). LWT Food Sci. Technol..

[69] Zilic S., Delic N., Basic Z., Ignjatovic-Micic D., Jankovic M., Vancetovic J. (2015). Effects of Alkaline Cooking and Sprouting on Bioactive Compounds, Their Bioavailability and Relation to Antioxidant Capacity of Maize Flour. J. Food Nutr. Res..

[70] Huang X., Cai W., Xu B. (2014). Kinetic Changes of Nutrients and Antioxidant Capacities of Germinated Soybean (Glycine Max l.) and Mung Bean (*Vigna Radiata* L.) with Germination Time. Food Chem..

[71] Nkhata S.G., Ayua E., Kamau E.H., Shingiro J.B. (2018). Fermentation and Germination Improve Nutritional Value of Cereals and Legumes through Activation of Endogenous Enzymes. Food Sci. Nutr..

[72] Fuliaş A., Vlase G., Vlase T., Oneţiu D., Doca N., Ledeţi I. (2014). Thermal Degradation of B-Group Vitamins: B1, B2 and B6: Kinetic Study. J. Therm. Anal. Calorim..

[73] Young H., Guk I., Myoung T., Sik K., Sik D., Hyun J., Joong D., Lee J., Ri Y., Sang H. (2012). Chemical and Functional Components in Different Parts of Rough Rice (*Oryza Sativa* L.) before and after Germination. Food Chem..

[74] Alamprese C., Ratti S., Rossi M. (2009). Effects of Roasting Conditions on Hazelnut Characteristics in a Two-Step Process. J. Food Eng..

[75] Stuetz W., Schlörmann W., Glei M. (2016). B-Vitamins, Carotenoids and Tocopherols in Nuts. Food Chem..

[76] Zhang Y.Y., Stockmann R., Ng K., Ajlouni S. (2020). Revisiting Phytate-Element Interactions: Implications for Iron, Zinc and Calcium Bioavailability, with Emphasis on Legumes. Crit. Rev. Food Sci. Nutr..

[77] Konietzny U., Greiner R. (2003). PHYTIC ACID| Properties and Determination. Am. J. Med. Sci.

[78] Castro-Alba V., Lazarte C.E., Perez-Rea D., Carlsson N.G., Almgren A., Bergenståhl B., Granfeldt Y. (2019). Fermentation of Pseudocereals Quinoa, Canihua, and Amaranth to Improve Mineral Accessibility through Degradation of Phytate. J. Sci. Food Agric..

[79] Inyang C.U., Zakari U.M. (2008). Effect of Germination and Fermentation of Pearl Millet on Proximate Chemical and Sensory Properties of Instant “Fura”—A Nigerian Cereal Food. Pak. J. Nutr..

[80] García-Mantrana I., Monedero V., Haros M. (2014). Application of Phytases from Bifidobacteria in the Development of Cereal-Based Products with Amaranth. Eur. Food Res. Technol..

[81] Deshpande S.S., Salunke D.K. (2002). Grain legumes, seeds and nuts: Rationale for fermentation. Fermented grains legumes, seeds and nuts: A global perspective. FAO Agric. Serv. Bull..

[82] Frontela C., García-Alonso F.J., Ros G., Martínez C. (2008). Phytic Acid and Inositol Phosphates in Raw Flours and Infant Cereals: The Effect of Processing. J. Food Compos. Anal..

[83] Greiner R., Konietzny U. (2006). Phytase for Food Application Phytase for Food Application. J. Food Technol. Biotechnol..

[84] Panche A.N., Diwan A.D., Chandra S.R. (2016). Flavonoids: An Overview. J. Nutr. Sci..

[85] Adetuyi F.O., Ibrahim T.A. (2014). Effect of Fermentation Time on the Phenolic, Flavonoid and Vitamin C Contents and Antioxidant Activities of Okra (Abelmoschus Esculentus) Seeds. Niger. Food J..

[86] Hur S.J., Lee S.Y., Kim Y.C., Choi I., Kim G.B. (2014). Effect of Fermentation on the Antioxidant Activity in Plant-Based Foods. Food Chem..

[87] Chaaban H., Ioannou I., Chebil L., Slimane M., Gérardin C., Paris C., Charbonnel C., Chekir L., Ghoul M. (2017). Effect of Heat Processing on Thermal Stability and Antioxidant Activity of Six Flavonoids. J. Food Process. Preserv..

[88] Modgil R., Sood P. (2017). Effect of Roasting and Germination on Carbohydrates and Anti-Nutritional Constituents of Indigenous and Exotic Cultivars of Pseudo-Cereal (Chenopodium). J. Life Sci..

[89] Ali M.A.M., Tinay A.H., Tinay E.l., Abdalla A.H. (2003). Effect of Fermentation on the in Vitro Protein Digestibility of Pearl Millet. Food Chem..

[90] Shimelis E.A., Rakshit S.K. (2007). Effect of Processing on Antinutrients and in Vitro Protein Digestibility of Kidney Bean (*Phaseolus Vulgaris* L.) Varieties Grown in East Africa. Food Chem..

[91] Kunyanga C.N., Imungi J.K., Okoth M., Momanyi C., Biesalski H.K., Vadivel V. (2011). Antioxidant and Antidiabetic Properties of Condensed Tannins in Acetonic Extract of Selected Raw and Processed Indigenous Food Ingredients from Kenya. J. Food Sci..

[92] Abdelhaleem H., Tinay A.H., El Mustafa A.I., Babiker E.E. (2008). Effect of Fermentation, Malt-Pretreatment and Cooking on Antinutritional Factors and Protein Digestibility of Sorghum Cultivars. Pak. J. Nutr..

